# Gamification and Oral Health in Children and Adolescents: Scoping Review

**DOI:** 10.2196/35132

**Published:** 2024-04-04

**Authors:** Rui Moreira, Augusta Silveira, Teresa Sequeira, Nuno Durão, Jessica Lourenço, Inês Cascais, Rita Maria Cabral, Tiago Taveira Gomes

**Affiliations:** 1 School of Medicine and Biomedical Sciences Instituto de Ciencias Biomédicas Abel Salazar University of Porto Porto Portugal; 2 Department of Stomatology and Oral Maxillofacial Surgery University Hospital Center of Santo António Porto Portugal; 3 Faculty of Health Sciences Fernando Pessoa University University of Porto Porto Portugal; 4 Institute for Research, Innovation and Development Fernando Pessoa Foundation University of Porto Porto Portugal; 5 Center for Innovative Biomedicine and Biotechnology University of Coimbra Coimbra Portugal; 6 Paediatrics Department, Maternal & Child Center of the North University Hospital Center of Santo António Porto Portugal; 7 Department of Community Medicine, Information and Decision in Health Medical School of Porto University of Porto Porto Portugal

**Keywords:** gamification, mechanisms of gamification, gamification components, intrinsic and extrinsic motivators, oral health care, health behavior, oral health care applications

## Abstract

**Background:**

Oral health is a determinant of overall well-being and quality of life. Individual behaviors, such as oral hygiene and dietary habits, play a central role in oral health. Motivation is a crucial factor in promoting behavior change, and gamification offers a means to boost health-related knowledge and encourage positive health behaviors.

**Objective:**

This study aims to evaluate the impact of gamification and its mechanisms on oral health care of children and adolescents.

**Methods:**

A systematic search covered multiple databases: PubMed/MEDLINE, PsycINFO, the Cochrane Library, ScienceDirect, and LILACS. Gray literature, conference proceedings, and WHOQOL internet resources were considered. Studies from January 2013 to December 2022 were included, except for PubMed/MEDLINE, which was searched until January 2023. A total of 15 studies were selected following PRISMA (Preferred Reporting Items for Systematic Reviews and Meta-Analyses) guidelines. The eligibility criteria were peer-reviewed, full-text, and empirical research related to gamification in oral health care, reports of impact, and oral health care outcomes. The exclusion criteria encompassed duplicate articles; unavailable full texts; nonoriginal articles; and non–digital game–related, non–oral health–related, and protocol studies. Selected studies were scrutinized for gamification mechanisms and outcomes. Two main questions were raised: “Does gamification in oral health care impact oral health?” and “Does oral health care gamification enhance health promotion and literacy?” The PICO (Patient, Intervention, Comparison, Outcome) framework guided the scoping review.

**Results:**

Initially, 617 records were obtained from 5 databases and gray literature sources. After applying exclusion criteria, 15 records were selected. Sample size in the selected studies ranged from 34 to 190 children and adolescents. A substantial portion (11/15, 73%) of the studies discussed oral self-care apps supported by evidence-based oral health. The most clearly defined data in the apps were “brushing time” (11/11, 100%) and “daily amount brushing” (10/11, 91%). Most studies (11/15, 73%) mentioned oral health care behavior change techniques and included “prompt intention formation” (11/26, 42%), “providing instructions” (11/26, 42%), “providing information on the behavior-health link” (10/26, 38%), “providing information on consequences*”* (9/26, 35%), “modeling or demonstrating behavior” (9/26, 35%), “providing feedback on performance” (8/26, 31%), and “providing contingent rewards” (8/26, 31%). Furthermore, 80% (12/15) of the studies identified game design elements incorporating gamification features in oral hygiene applications. The most prevalent gamification features were “ideological incentives” (10/12, 83%) and “goals” (9/16, 56%), which were found in user-specific and challenge categories, respectively.

**Conclusions:**

Gamification in oral health care shows potential as an innovative approach to promote positive health behaviors. Most studies reported evidence-based oral health and incorporated oral health care behavior change techniques.

## Introduction

People’s health behaviors, such as physical activity, diet, tobacco and alcohol use, recreational drug consumption, and adherence to chronic medications, directly influence their health risks and consequent diseases. To decrease the burden of preventable chronic diseases and enhance well-being in society, it is essential to bring about a change in behavior [1-4]. According to the World Health Organization, oral health is a strong indicator of general health, well-being, and quality of life. The treatment of oral pathologies is expensive and usually not covered under universal health care, accounting for 5% of health care expenditures and 20% of out-of-pocket expenses in wealthy countries [5-11]. Health care systems have limited resources, and making informed decisions based on data collection, focusing on individuals, can provide better health outcomes without incurring additional costs; this approach can deliver better value and at the same time reduce costs [12,13].

Motivation is a core target of a wide range of established behavior change techniques [1,14-17]. Computer games can be used to increase health-related knowledge and promote desirable health behaviors in children [18]. Games are designed to provide enjoyment, engagement, and satisfaction [1,19-22]. Mobile phones and mobile health technologies can address these issues at low costs [1,23,24]. Mobile devices are useful for delivering health interventions due to their widespread adoption, powerful technical capabilities, and portability [25]. The positive emotional attachment with the user may increase the benefit of health promotion via mobile devices, allowing health interventions to be delivered immediately, anytime, and anywhere [26]. The use of health care apps provides easy access to information and has the potential to improve patient engagement and treatment compliance [27]. Indeed, the number of health care apps available has been growing year after year, with over 200 billion app downloads worldwide from the Apple App Store and Google Play in 2020 [28].

Bohn et al [29] found that educational applications are valuable tools for enhancing patient-provider communication in dental settings. Studies have pointed out that the traditional educational approach, which relies mainly on reading and listening to standardized content, should be replaced with customizable and interactive involvement, using communication tools that are familiar to newer generations [30]. Gamification is a possible response to overcoming the challenges of communication and motivation in health care [31], as it can track individual behaviors and involve users in goal-chasing activities while displaying progress and feedback through personalized information apps [1,32].

Deterding et al [33] define gamification as the integration of game design elements into nongame contexts [34]. This process enriches products, services, and information systems with game design features to positively influence the motivation, productivity, and behavior of users [33,35,36]. Gamified systems commonly use motivational features such as immediate success feedback (reward mechanism), continuous progress feedback, and goal setting. These systems work through interface elements such as point-scoring systems, badges, levels, challenges, competitions, relatedness support, social feedback (engagement loops), recognition, comparison through leaderboards, teams, communication functions, autonomized support through customizable avatars and environments, and narratives providing emotional and value-based rationales for certain activities (customization) [1,34,37,38]. Users receive badges that represent success and can be shared in social networks and displayed in a digital trophy cabinet when new milestones are achieved [35]. Recently, the self-determination theory has become a key framework for health behavior interventions and research [1,39-43]. According to this theory, gamification induces 4 main intrinsic motivators: user satisfaction, conveyance of optimism, provision of meaning [35,44], and facilitation of social interactions [45,46].

Apart from financial aspects, extrinsic motivators systematically activate intrinsic motivators, such as social recognition, support of learning processes, and behavioral change. The evidence suggests that positive outcomes are stronger when gamification is used to target behavioral outcomes [1,23,24,47-50]. However, critics have pointed out the lack of high-quality effect studies on gamification [22,38,51,52]. Nevertheless, clinicians have an opportunity to promote engagement in health promotion through a motivating, fulfilling, and fun activity [53]. Therefore, this study aims to analyze the impact of gamification and its mechanisms on oral health care.

The objective of this scoping review was to analyze the impact of gamification and its mechanisms on oral health care in children and adolescents. We assessed the effectiveness of gamification in promoting changes in oral health behavior and enhancing oral health care outcomes for its users. Furthermore, we investigated the role of gamification in oral health care, including the integration of evidence-based oral health care concepts and gamification design elements in application design.

## Methods

### Objective

This scoping review aims to analyze the impact and efficiency of gamification mechanisms on oral health care, with a focus on promoting user engagement to expand oral health literacy and support oral health care policies, in accordance with PRISMA (Preferred Reporting Items for Systematic Reviews and Meta-Analyses) guidelines [54,55].

### Scoping Review

This review used a systematic methodology to identify gamification on oral health care apps, evaluate their features, identify their gamification mechanisms, and follow their outcomes.

We did not apply any restrictions related to population samples or oral health care systems, but we excluded contexts outside oral health.

The following main questions guided our analysis:

- Does gamification in oral health care impact oral health?
- Does oral health care gamification enhance health promotion and literacy?

On the basis of these questions, we searched several domains, including oral health care outcomes, gamification design elements, evidence-based oral health care concepts used in app development, and oral health behavior changes related to gamification. We use the PICO (Patient, Intervention, Comparison, Outcome) framework to elucidate the systematic review questions:

- P (Patient, Population, or Problem): the population under investigation included children and adolescents: mother-preschooler (3-6 years old) dyads and adolescents (younger than 16 years).
- I (Intervention): the intervention under investigation was the gamification strategy and its usefulness for oral health promotion and literacy.
- C (Comparison): gamification was compared with its alternative, traditional learning methods.
- (Outcome): the outcome assessed was divided into 2 main sections: behavior change techniques and gamification mechanisms.

### Search Strategy

The search was conducted across several electronic bibliographic databases, including PubMed/MEDLINE, PsycINFO, the Cochrane Library, ScienceDirect, and LILACS. In addition, gray literature, conference proceedings, and WHOQOL internet resources were assessed. The search strategy included terms related to gamification and oral health care such as gamification, oral health care, policies, games, digital, apps, and outcomes. The authors used a controlled and hierarchically organized vocabulary produced by the National Library of Medicine called the Medical Subject Headings to ensure that the search results accurately reflected the subject content of journal articles as they are published. The search strategy enabled us to identify both published and unpublished studies. All sources were last searched until July 2023, except for PubMed/MEDLINE, which was searched until January 2023. The references to gamification date back to nearly 2010, and articles exploring gamification in oral health were only mentioned or studied in the last decade. There were no language restrictions, and studies published between January 2013 and December 2022 (10 years) were included.

The search string used on PubMed was “Gamification” [mh] OR “oral healthcare” [tiab] OR “gamification” [tiab] OR ad [tiab] OR “applications” [tiab] OR “gamification” [tiab] OR “digital” [tiab] OR “games” [tiab] OR “outcomes” [mh] OR policies [tiab] OR gamification [tiab] OR “oral health” [mh:noexp] (see Multimedia Appendix 1).

Initially, all types of articles were considered eligible, including systematic reviews, research articles, and prospective and retrospective studies, as long as they met the following criteria: (1) peer-reviewed, (2) full-text papers, (3) empirical research (qualitative and quantitative), (4) explained research methods, (5) gamification as a research subject, (6) effect reported in terms of impact (affect, behavior, and cognition) and user experience, and (7) oral health care outcomes. Criteria 1 to 4 were implemented to ensure focus on high-quality work reporting original research. Criteria 3, 4, and 7 were also included to enable assessment of the quality of evidence. Criterion 5 admitted papers that studied gamification in a broader concept, even if it did not elicit game elements. Criteria 6 and 7 were chosen to assess reported health and well-being outcomes and potential mediators, with user experience being included given its prevalence as an outcome.

However, studies were excluded if they (1) were duplicated; (2) had full text not available; (3) were not the original article; (4) did not refer to a game; (5) were nondigital, such as conventional games like cards or board games; (6) did not concern oral health; and (7) were study protocols without outcomes to report.

### Study Quality Assessment, Data Extraction, and Analysis Plan

All searched articles were filtered using broad selection criteria framed as questions:

- Does gamification in oral health care impact oral health?
- Does oral health care gamification enhance health promotion and literacy?

The study selection and data extraction were performed blindly. After the search, all references were imported into a reference management system (Mendeley), and duplicates were removed. The remaining articles’ titles and abstracts were assessed to identify eligible studies. To determine eligibility, three additional questions were asked and answered: (1) Is the topic relevant to the defined scope? (2) Does it meet the inclusion and exclusion criteria? and (3) Is the methodology appropriate? To ensure a comprehensive, transparent, and objective extraction process, a standardized prepiloted form was used to extract data from the included studies. Two reviewers independently extracted the data, and any discrepancies were resolved through discussion with a third author. Additionally, the third reviewer further scrutinized the data to verify the consistency of the extraction process and resolved any remaining discrepancies. In case of missing or additional data, other researchers were contacted [56,57].

The eligibility assessment of each full-text paper was conducted by 2 independent raters. In cases where discrepancies arose, they were resolved through discussion and comparison of our evaluations. Articles were excluded from our review when both investigators unanimously concurred on their ineligibility due to inappropriate methodology or results that did not address the key research questions at hand. This rigorous and collaborative approach to eligibility assessment ensured the quality and relevance of the articles included in our study.

Each article was classified as having low, moderate, or high relevance. Articles were deemed highly relevant if they effectively demonstrated an impact on the considered items, whereas moderate relevance was attributed to those that projected such items. Low relevance was assigned to manuscripts that did not present any conclusions or perspectives in these domains.

Data from the scoping literature were extracted into an Excel (Microsoft Corp) data sheet using a support checklist. The data sheet was divided into sections dedicated to a theory, area, concept, theme, or element from the framework of gamification and oral health care, including game mode, population sample, gamification components, behavior change techniques, and outcomes. After synthesizing the data and assessing the quality of the evidence, the writing of the scoping review article began.

## Results

### Process Selection

**Figure 1:**
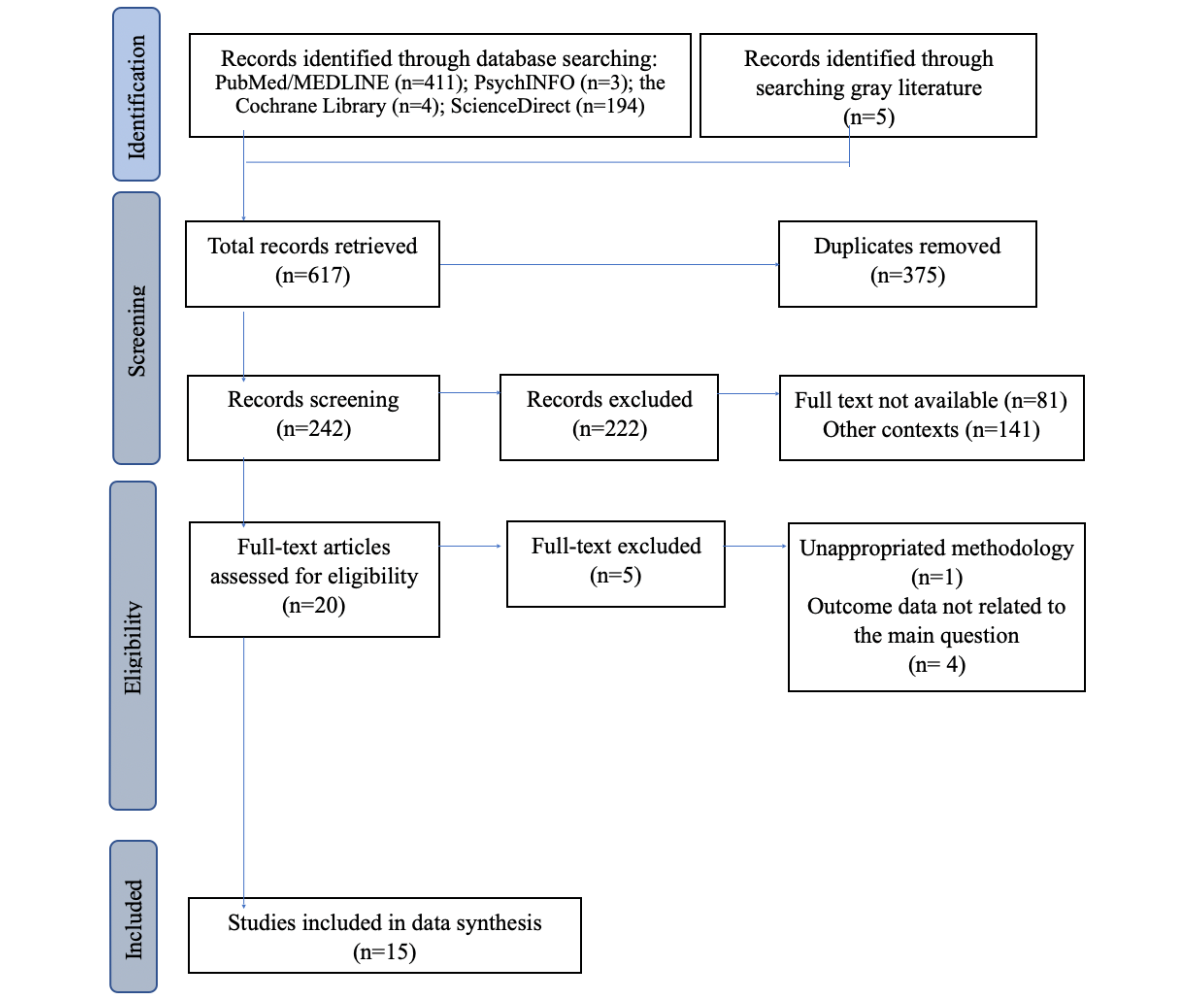
PRISMA (Preferred Reporting Items for Systematic Reviews and Meta-Analyses) flow diagram.

Figure 1 shows the PRISMA flowchart outlining the process of record identification, selection, eligibility, and inclusion. Initially, 617 records were retrieved from 5 databases and gray literature sources. After removing duplicates and records that did not meet the inclusion criteria, 15 records were selected for analysis.

### Sample Characteristics

Of the 15 studies available (see Multimedia Appendix 2 [9,18,26,27,30,58-67]), the majority (n=8, 53%) collected data at multiple time points (2 or more) from various points or conditions [18,30,58-62,68]. Of these 8 studies, 7 were blind, randomized control trials [18,30,58,59,61-63]. A content analysis of the applications for behavior change was adopted in 7 studies [9,27,60-62,64,65]. One-time cross-sectional questionnaires were applied in 3 studies [26,66,67].

The sample size in the selected studies ranged from 34 to 190 individuals. All participants were children younger than 13 years, except for 4 studies that also included adults [26,61,62,65]. Mobile apps were the predominant modality used to change oral health care behavior in the studies (n=12), with 3 exceptions based on computer games. One of the games was available on a tablet and DVD for PC [63], whereas the other 2 apps were associated with a toothbrush sensor [58,59].

### Oral Hygiene Evidence-Based Categories

**Figure 2:**
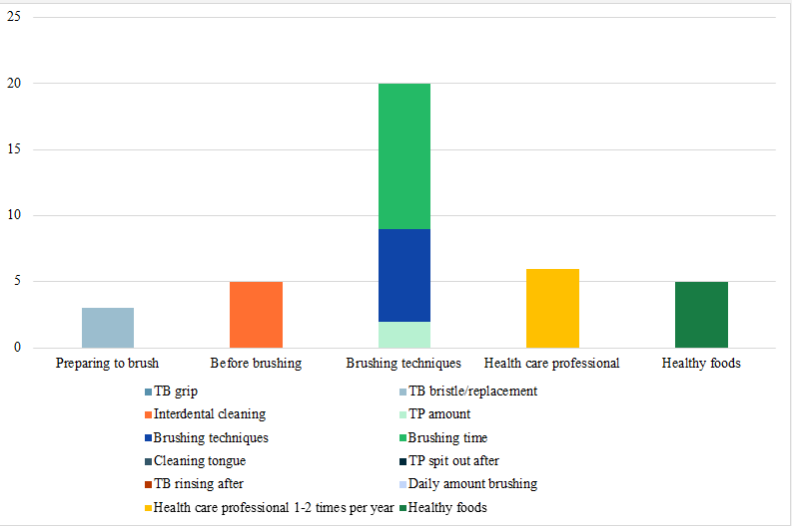
Evidence-based content related to group categories and archetypes of oral hygiene, as recognized in the chosen studies. The y-axis displays the listed group categories and archetypes, whereas the x-axis signifies the frequency of their occurrence within the studies. The accompanying legend elucidates the color-coding system denoting distinct group categories and archetypes. TB: toothbrush; TP: toothpaste.

The oral self-care applications supported by evidence-based oral health were mentioned in 73% (11/15) of the selected studies. The group categories and archetypes of evidence-based content related to oral hygiene were identified by analyzing the selected studies, as illustrated in Figure 2.

We found that the most clearly defined types of information in the applications were “brushing time” for at least 2 minutes (11/11, 100%) and “daily amount brushing” at least twice a day (10/11, 91%). However, “toothpaste spit out after” brushing was only found in 1 application (1/12, 8%), and “cleaning the tongue” and “toothbrush grip” were never mentioned.

### Behavior Change Techniques

Behavior change techniques for oral health care resulted from mechanisms of gamification, either implicit or explicit. Oral health care behavior change techniques were mentioned in 73% (11/15) of the studies. The data collected from these studies were used to create a behavior change score with 26 items [9]. The studies were analyzed to determine the frequency of behavior change techniques used in oral health applications, as shown in Table 1.

Among the behavior change techniques scrutinized, a set of 7 distinct components emerged as the most prevalently used across the array of surveyed studies. These components, along with their respective frequencies of use, are outlined as follows: “prompt intention formation” (11/26, 42%), “provide instructions” (11/26, 42%), “provide information on behavior-health link” (10/26, 38%), “provide information on consequences” (9/26, 35%), “model or demonstrate behavior” (9/26, 35%), “provide feedback on performance” (8/26, 31%), and “provide contingent rewards” (8/26, 31%).

**Table 1:** Frequency distribution of behavior change techniques observed in oral health applications identified in the selected studies (N=137).

Behavior change techniques	Value, n (%)
1. Provide information behavior health link	10 (7.3)
2. Provide information on consequences	8 (6.6)
3. Provide information seeking others’ approval	1 (0.7)
4. Prompt intention formation	11 (8)
5. Prompt barrier identification	3 (2.2)
6. Provide general encouragement	7 (5.1)
7. Set graded tasks	2 (1.5)
8. Provide instruction	11 (8)
9. Model or demonstrate behavior	9 (6.6)
10. Prompt specific goal setting	4 (2.9)
11. Prompt review of the behavioral goals	5 (3.6)
12. Prompt self-monitoring behavior	2 (1.5)
13. Provide feedback on performance	8 (5.8)
14. Provide contingent rewards	8 (5.8)
15. Teach to use prompts or cues	4 (2.9)
16. Agree on behavioral contract	7 (5.1)
17. Prompt practice	7 (5.1)
18. Use follow-up prompts	4 (2.9)
19. Provide social comparison	2 (1.5)
20. Plan social support or change	2 (1.5)
21. Prompt identification as a role model	2 (1.5)
22. Prompt self-talk	2 (1.5)
23. Relapse prevention	4 (2.9)
24. Stress management	1 (0.7)
25. Motivational interviewing	5 (3.6)
26. Time management	7 (5.1)

### Gamification Mechanisms

The game design elements based on the gamification features rating criteria for oral hygiene applications were recognized in 12 (80%) of the 15 studies. Table 2 illustrates the 26 gamification features [9] that were considered, along with the number of times each element was identified.

On average, the applications included in the study possessed an average of 10.6 of 31 potential gamification features. Notably, specific game design elements were prevalently used within various categories. Among the system design features, “meaning” (10/36, 28%) stood out prominently. Within the challenges category, “goals” (9/16, 56%) emerged as the most frequently incorporated element. For rewards, “ownership” (9/22, 41%) was notably prevalent. Among social influences, both “collaboration” and “reputation” (both 3/19, 16%) were prominent. Additionally, within the user-specific category, “ideological incentives” (10/12, 83%) exhibited a substantial presence. Interestingly, none of the applications used “badges,” “conforming behavior,” “virtual goods,” or “self-expression” (Table 2).

**Table 2:** Frequency distribution of game design elements detected in the surveyed studies.

Game design elements		Value, n (%)
**System design (n=36)**		
	Visual feedback	9 (25)
	Audible feedback	4 (11)
	Reminder	5 (14)
	Meaning	10 (28)
	Integration concepts	3 (8)
	Visually resembling games	1 (3)
	Fantasy	4 (11)
**Challenges (n=16)**		
	Goals	9 (56)
	Time pressure	4 (25)
	Progressive disclosure	3 (19)
**Rewards (n=22)**		
	Ownership	9 (41)
	Achievement	5 (23)
	Point system	4 (18)
	Badges	0 (0)
	Bonus	4 (18)
**Social influences (n=19)**		
	Loss aversion	2 (11)
	Status	2 (11)
	Collaboration	3 (16)
	Reputation	3 (16)
	Competition	1 (5)
	Envy	1 (5)
	Shadowing	2 (11)
	Social facilitation	2 (11)
	Conforming behavior	0 (0)
	Leaderboards	2 (11)
	Altruism	1 (5)
	Virtual goods	0 (0)
**User specifics (n=12)**		
	User levels	1 (8)
	Ideological incentives	10 (83)
	Virtual characters	1 (8)
	Self-expression	0 (0)

## Discussion

### Principal Findings

The oral health outcomes related to gamification interventions highlighted the role of gamification in promoting oral health care and literacy. This scoping review also highlights the limitations of currently available oral health care apps and points out the main areas to invest in for the future. A total of 11 (73%) of the 15 articles found positive impacts of using oral health apps, especially in children and adolescents. They facilitate the responsiveness of oral preventive care [66]; improve knowledge in high-risk populations; encourage dietary changes [63]; and promote a reduction of clinical plaque, gingival, and caries indexes [26]. Additionally, they show a statistically significant improvement in health care indices [58], tooth brushing quality (duration and distribution) [60], and motivation to brush teeth for longer [26] and seem effective in adolescents with fixed orthodontic appliances by self-reported behavior and psychosocial factors [59]. Gamification structures augment oral health literacy, facilitate user alertness for oral health care themes and professional feedback, and engage commitment. A greater improvement in gingival status is commonly reported [27,61,62,69].

The feedback provided by participants showed a higher level of satisfaction in learning about oral health care through games rather than traditional noninteractive methods. Most studies reported a positive impact of gamification, particularly in children and adolescents, who are considered the main target audience of these apps [18,26,30,58-66].

The studied apps contained educational content with evidence-based dentistry and high-quality teaching for oral self-care. Some of these also feature gamification elements and behavior change techniques. The results of the studies demonstrate that these apps have excellent functionality, effectiveness, efficiency, and user satisfaction [9,64,66]. Several studies evaluating multiple oral hygiene apps have found evidence-based content, such as brushing time and daily amount of brushing. Fijačko et al [9], Parker et al [27], and Hotwani et al [64] all reported on these elements.

The health behavior change techniques found in the analyzed apps included prompt intention formation, shaping and demonstrating behavior, providing information about the link between behavior and health consequences, instructions, and contingent rewards [26,30,58,63,66,67]. A set of 7 distinct components emerged as the most prevalently employed across the array of surveyed studies. These components, along with their respective frequencies of use, are outlined as follows: “prompt intention formation,” “provide instructions,” “provide information on behavior-health link,” “provide information on consequences,” “model or demonstrate behavior,” “provide feedback on performance,” and “provide contingent rewards.” These components collectively represent the core elements of behavior change techniques that were consistently integrated into the analyzed oral health applications, aiming to enhance engagement and promote positive behavior change.

Regarding game design elements, these applications emphasized feedback, goal attainment, sense of ownership, and ideological incentives [27,61,65-67]. Parker et al [27] identified some recurring game design elements among 20 apps analyzed, such as knowledge provision, self-monitoring of frequency, and duration of toothbrushing. Hotwani et al [64] found that information provision, goal setting, feedback, progressive disclosure, and time pressure were frequently used in the 6 apps evaluated. Fijačko et al [9] analyzed 17 apps and identified time pressure, digital characters, and fantasy as key game design elements.

### Comparison With Prior Work

Delivering trustworthy information to users is essential for promoting healthy habits. Health care apps should undergo validation by health care institutions and professionals before becoming public to ensure their accuracy and reliability. However, there is a risk of users becoming overly dependent, potentially compromising the need for regular appointments with oral health care professionals in real life [27,70].

Considering evidence-based oral health care, most apps emphasize brushing for at least 2 minutes and twice a day. Although this is an important core of oral hygiene recommendations, there is still room for improvement. To achieve holistic oral care, it would be advisable to incorporate other aspects such as oral hygiene techniques, the use of devices, dietary advice, sugar intake control, guidance on early childhood caries, baby oral hygiene, the effects of fluoride, the use of fluoride toothpaste, toothbrushing training videos, and regular dental visits. The development of apps should be based on theoretical models when designing educational content, and the accuracy of the content should be a priority to bring about real behavior change [61,64,70]. Sharif and Alkadhimi [65] went beyond the basics and included interdental cleaning, spitting out after brushing, avoiding mouth rinsing after brushing, characteristics of the toothbrush, and the quantity of fluoride content in toothpaste and mouthwash. Other advisable strategies include reporting about others’ approval, social interactions with oral health professionals and other application users, identifying barriers to oral hygiene and potential overcoming strategies, providing encouragement, setting graded tasks and goals, displaying tracked data and objectives, feedback on performance, setting a behavioral contract with oral health professionals, social comparison, and social support [1,9,27,35,63,65,71].

Patient adherence to a smartphone app is more effective because of the ability to customize reminders and prompts, constant accessibility, adjustability to the user, ability to provide tailored feedback, widespread use, and interactive features [63,72,73].

### Strengths and Limitations

Despite the proven efficiency of gamification in health care promotion and prevention, it remains an unexplored territory in oral health, mainly applied to specific educational purposes and oral health promotion [74]. This scoping review highlights the limitations of currently available oral health care applications and points out the main areas to invest in for the future. Two major limitations were found in this study. The first is the limited availability of articles related to the main topics. Gamification, within the context of health care, is not a recent concept. However, its quantitative assessment, particularly in the field of oral health, remains relatively uncommon. To address such limitation and ensure a comprehensive review, we diligently accessed and explored 5 databases, along with incorporating gray literature sources. This approach was essential to include as many relevant studies as possible, aligning with our predefined inclusion criteria. Considering the diverse nature of our search strategy, we believe that we have made every effort to provide a reliable representation of the existing literature in the field of gamification in oral health care.

The second limitation is the heterogeneous studies with varying focus and dispersing attention. The diversity of approaches makes a rigorous comparison more challenging.

Taking into account the multiple aspects involved in gamification strategies and by transparently outlining these parameters, we believe that our work can serve as a valuable reference for future researchers seeking to design studies that address and overcome these challenges. Our hope is that this will pave the way for a more effective understanding of the underlying mechanisms behind the implementation of gamification in oral health context.

### Future Directions

Future studies could focus on other age groups as well, such as the study by Zolfaghari et al [61], which developed applications for mothers of children, improving their oral health literacy and practice and promoting plaque control in children within just 1 month of use [61,74,75].

There is potential for future optimization of key gamification features, such as badges, encouragement of correct behavior, digital goods, self-expression, reminders, fantasy themes, time pressure, disclosure of progress, achievements, points systems, bonuses, loss aversion, status, collaboration, reputation, competition, shadowing, social facilitation, leaderboards, altruism, user levels, and digital characters [26,27,30,66,71,76].

### Conclusions

Gamification in oral health care does have an impact; it enhances oral health promotion and literacy. It represents a potential new approach for oral health care providers to change people’s oral health behavior. The most frequent game design mechanisms adopted were meaning, ideological incentives, feedback, goals, and ownership. Some authors have highlighted several factors for gamification success, including engagement strategy, applications aesthetics, evidence-based information content, behavioral change taxonomies, attention to psychological needs, evaluation, validation, quality assessment, and professional regulation standards for oral health care applications. More studies are needed to better understand the clinical, psychological, and social processes involved in selecting the most efficient gamification mechanisms. The process of mobile health in oral health care is in the initial stage, but gamification is crucial for improving individual health-related practices.

## Appendix

Multimedia Appendix 1 Search string.

Multimedia Appendix 2 Full paper details.

Multimedia Appendix 3 PRISMA-ScR checklist.

## Abbreviations

PICO: Patient, Intervention, Comparison, Outcome
PRISMA: Preferred Reporting Items for Systematic Reviews and Meta-Analyses
