# Supplementary figures and images for: Gamification and Oral Health in Children and Adolescents: Scoping Review
Source: Interact J Med Res. 2024 Apr 4;13:e35132. doi: 10.2196/35132 (PMC11027059; doi:10.2196/35132)

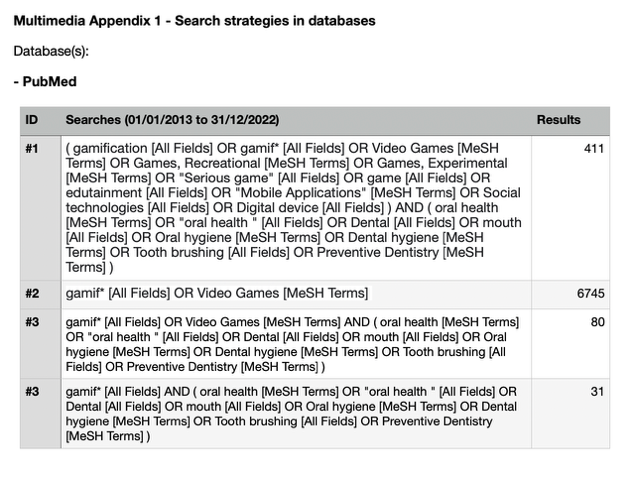

Supplement: Multimedia Appendix 1 [file ijmr_v13i1e35132_app1.png]
