# Supplementary material for: Gamification and Oral Health in Children and Adolescents: Scoping Review
Source: Interact J Med Res. 2024 Apr 4;13:e35132. doi: 10.2196/35132 (PMC11027059; doi:10.2196/35132)
Supplement: Multimedia Appendix 2 [file ijmr_v13i1e35132_app2.docx]

Multimedia Appendix 2: Full paper details

| **PMID**  **Author** | **Game Mode** | **Population sample** | **Gamification components** | **Oral hygiene categories** | **Behavior changes techniques** | **Outcomes** |
| --- | --- | --- | --- | --- | --- | --- |
| **Paper 1** [[66]](https://paperpile.com/c/d0fCA1/Ehgf)  Ito et al, 2013 | Serious game | Children  115 children  3-6 years  CSQ | Goal: tooth remains whole.  Evaluation of avatar oral state |  | Evaluate the advantages of decision-making | Facilitate the awareness of preventive dental care for children aged 3 to 6 years |
| **Paper 2** [[18]](https://paperpile.com/c/d0fCA1/Zx5q)  Panic et al*,* 2014 | Interactive game | Children  190 children  7-9 years  BRCT | Scores: removing residues.  Avatar Beaver Ben teach.  Threat weak/strong.  Questionnaire.  Snack reward  (unhealthy/healthy) | Info oral hygiene; Brushing teeth properly.  Dentist 2xyear.  Healthy foods. |  | Threat message + health information; medium in which information is presented influences the effectiveness of the message.  Games may not always be the most effective medium to reinforce a strong threat appeal |
| **Paper 3** [[26]](https://paperpile.com/c/d0fCA1/ZqFo)  Underwood et al*,* 2015 | App  ‘Brush DJ’ | Children and adults  189 responds  CSQ | Quiz  motivate users to brush for two minutes by playing music.  Reminder: brush, appointments  Information: oral hygiene | Floss and interdental brushes before.  Brushing teeth for 2 min, twice a day.  brush different parts of the mouth visually, by vibration and sound after 30‘’;  not rinse after.  mouthwash at different time  appointments.  Change toothbrush 3 months.  Video tutorials. | Instructions and  Demonstration: animated videos.  Prompts: interdental before brushing, not rinse after.  Cues: brush different parts visually, vibration and sound after 30‘’  Social reward: share via social media the name of the song that you have listened.  Other reward: smile and applause with 2-min brushing | 70% of their teeth felt cleaner.  80% motivated to brush their teeth for longer.  92.3% would recommend the app to their friends and family.  How the app helped toothbrushing: motivation, education, compliance, and perceived benefit |
| **PMID**  **Author** | **Game Mode** | **Population sample** | **Gamification components** | **Oral hygiene EB categories** | **Behavior changes techniques** | **Outcomes** |
| **Paper 4** [[30]](https://paperpile.com/c/d0fCA1/5oUH)  Zotti et al*,* 2016 | App  ‘Brush Game’ | Children  80 adolescents with multibrackets  Mean age:14.1 years  Duration: 1 year  BRCT | WhatsApp competit  2 selfies(b/a)/week.  Nickname; reminder  Rank: best 5/week  Score: plaque index, gingival index, white spots, caries  Social motivation  Share information.  Share videos | Video tutorials.  Brush teeth.  Oral hygiene kit: toothpaste, toothbrush, mouthwash, interproximal brush, dental floss, plaque disclosing tablets | NA | Significantly lower values of both plaque and gingival indexes; lower incidence of new white spots and caries, compared with the control group. |
| **Paper 5** [[63]](https://paperpile.com/c/d0fCA1/OzRH)  Aljafari et al*,* 2017 | EducationComputer game; Tablet and DVD | Children and parents  4-10 years  109 families  Duration: 3 months  BRCT | Pictorial dietary quiz;  Children's dietary questionnaire;  Self reporting snacking and toothbrushing;  Scores | Education messages as DBOH  Follow up dental visit |  | Oral health education using a computer game can be as satisfactory and as effective in improving high-risk-children’s knowledge as one-to-one education. Lead to positive dietary changes. |
| **Paper 6** [[67]](https://paperpile.com/c/d0fCA1/fXnc)  Campos et al*,* 2019 | App  ‘1,2,3… Brush!’ | Children  43 Preschoolers  3-5 years  Observation and survey | Quiz  Animation and  Sound effects  Selection of a character;  Story menu;  Game: solving activities; task complete: yes/no | Caries, diet, oral hygiene, bottle feeding, habits, and malocclusion;  Postmeal oral hygiene; tooth brushing before bedtime; dental floss | Interactive children’s book flux design;  convey oral health information in a playful manner | Excellent results in effectiveness, efficiency, and satisfaction.  Dietary education was observed to be necessary for the children and their guardians. |
| **Paper 7** [[27]](https://paperpile.com/c/d0fCA1/fCje)  Parker et al*,* 2019 | 20 Apps | CABC  MARS | Motivation: knowledge provision;  Self-monitoring frequency and duration toothbrushing; | Provision of oral hygiene advice, ability to time toothbrushing, and gamification | None of the apps profiled clearly stated if they had been developed based on theoretical models of behavior change | Lack of professional regulation on apps is a risk of inaccurate information. Evaluation, validation, and quality assessment of health care apps is needed before recommending. |
| **PMID**  **Author** | **Game Mode** | **Population sample** | **Gamification components** | **Oral hygiene categories** | **Behavior changes techniques** | **Outcomes** |
| **Paper 8** [[58]](https://paperpile.com/c/d0fCA1/FFKW)  Alkilzy et al*,* 2019 | App and toothbrush sensor | Children  49 children  5-6 years  Duration: 12 weeks  BRCT | Oral hygiene indices:  -papillary bleeding;  -plaque  -adherence child;  Visualize and reward proper brushing in form and time;  learning progress displayed on smartphone and shared;  Bonus points (bears): data of each brushing session, optimal brushing | Step by step instructions brushing;  Replace toothbrushes in 3 months;  Brush 2× /day, 2 min;  Regular visits to dentist | DAYA system;  motivational method with manual toothbrush child friendly design, gravity sensor and app.  HAPA theory changes in psychosocial factors: outcome expectancies, intention, action self efficacy, coping planning, action control. | Statistically significantly better oral health indices were observed;  Enormous possibilities of a toothbrushing application via the smartphone, at least for medium-term oral hygiene improvement in preschool children |
| **Paper 9** [[60]](https://paperpile.com/c/d0fCA1/CzaA)  Jacobson et al*,* 2019 | App  ‘Brush Up’ and toothbrush sensor (and laptop) | Children  34 children  5-6 years  Duration: 14 days to 1 year  Collect info  CSQ | Teach brush along a song (and video) 3 min; rhythm sets pace strokes,  Reward: according or correction of errors;  Penalty: errors by verbal, visual and sound effect cues and score penalty | Modified Bass Stillman Technique;  Brush 3 min | Modeling, instructional song, and immediate performance feedback. | Can potentially improve toothbrushing quality (duration and distribution) in children; longer use, greater improvements |
| **Paper 10** [[65]](https://paperpile.com/c/d0fCA1/j72G)  Sharif and Alkadhimi, 2019 | 20 Apps | Children and adults  MARS  CABC |  | Interdental cleaning; Brush teeth 2 min 2× day (night, other), spit out after; fluoride toothpaste; fluoride containing mouth rinse daily (0.05% NaF) at a different time to brushing; toothbrush medium |  | Currently available apps appear to be functional;  Need to improve engagement, aesthetics and most importantly information content;  Evaluation, validation, and quality assessment of health care apps is essential. |
| **PMID**  **Author** | **Game Mode** | **Population sample** | **Gamification components** | **Oral hygiene categories** | **Behavior changes techniques** | **Outcomes** |
| **Paper 11** [[64]](https://paperpile.com/c/d0fCA1/lIZ9)  Hotwani et al*,* 2020 | 6 Apps  Ranking | Children  CABC |  | Tooth brushing  (CALO-RE taxonomy) | Information provision (general);  Goal setting (behavior);  Prompt practice;  Setting grade tasks, self monitoring of behavior outcome, demonstration of behavior, prompt use of imagery and time management | Very few apps have included behavior change taxonomies in their features; Desirable to focus on training based on guidelines; Emerging option for oral hygiene behavior change, address psychological needs; intrinsic motivation |
| **Paper 12** [[59]](https://paperpile.com/c/d0fCA1/1dlU)  Scheerman et al*,* 2020 | App  ‘The White Teeth’  Quiz of habits;  Selfie | Children  132 adolescents with multibrackets  12-16 years  Duration: 12 weeks  BRCT | Oral health info; automatic coaching program;  positive reinforcement;  feedback performance;  Push notifications;  Timer brushing;  Questionnaires;  Score: plaque, gingival index | Intervention mapping;  Brush teeth 2 min;  Interproximal brush;  Toothpick;  Dental floss;  Fluoride mouth rinse; | Health Action Process Approach (HAPA) theory:  risk perception; action, intention, maintenance and recovery self‐efficacy; action control and planning; coping planning, social influences; outcome expectancies. | Effective in reducing in adolescents with fixed orthodontic appliances dental plaque and marginal bleeding (primary outcomes), and self‐reported oral health behaviors and their psychosocial factors (secondary outcomes) |
| **Paper 13** [[9]](https://paperpile.com/c/d0fCA1/6ylo)  Fijacko et al*,* 2020 | 17 Apps | Children  Under 13 years  CABC  PICO criteria | Time pressure, virtual characters, fantasy.  (and audible feedback: songs)  less frequently: conforming behavior and leaderboards | Most frequent:  brushing time (2-3 minutes) | Quantified behavior scores (Behavior Change score, uMARS score, and Coventry, Aberdeen, and London-Refined [CALO-RE] score) | Educational content with evidence-based dentistry and high-quality background for oral self-care in children.  The majority of apps included gamification features and behavior change techniques to perform and maintain oral hygiene in children |
| **PMID**  **Author** | **Game Mode** | **Population sample** | **Gamification components** | **Oral hygiene categories** | **Behavior changes techniques** | **Outcomes** |
| **Paper 14** [[61]](https://paperpile.com/c/d0fCA1/6ylo)  Zolfaghari et al*,* 2021 | Gamified smartphone application *versus* simple app | 58 mother-preschooler dyads  Duration: 1 month | . | Information about early childhood caries, health diet, sugars, baby-oral hygiene, fluoride effect, fluoride toothpaste | Tooth-brushing training video and regular dental visits Plaque index (PI) of children | Mean knowledge score and mean practice score increased (both in simple app and gamified app) but more markedly in the gamified group. Children had better Plaque control in gamified app group (*P*<.05). |
| **Paper 15** [[62]](https://paperpile.com/c/d0fCA1/6ylo)  Shirmohammadi et al*,* 2022 | Gamified smartphone application *versus* educational pamphlet and verbal explanations | 51 mother-preschooler dyads  Duration: 1- to 3-month follow-up |  | Pediatric dentistry knowledge, attitude and practice regarding children's oral health | Modified plaque index (m-PI) and modified gingival index (m-GI) | Both trainings improved mothers' knowledge, practice regarding children's oral health and reduced children's m-PI and m-GI (*P*<.05). The 3-month follow-up revealed a better m-GI in application intervention group (*P*<.001). |

Legend:

**Paper number** [[number of the reference in the manuscript]](https://paperpile.com/c/d0fCA1/6ylo)

BRCT= Blinded Randomized Control Trials

CSQ= Cross Sectional Questionnaire

CABC= Content Analysis of the Apps for Behaviour Change
