# Supplementary material for: Gamification and Oral Health in Children and Adolescents: Scoping Review
Source: Interact J Med Res. 2024 Apr 4;13:e35132. doi: 10.2196/35132 (PMC11027059; doi:10.2196/35132)
Supplement: Multimedia Appendix 3 [file ijmr_v13i1e35132_app3.pdf]

**Multimedia Appendix 3 - PRISMA Checklist**

| Section | # | Checklist item |
|---|---|---|
| Title | 1 | Gamification in Oral Health - a Systematic Review |
| Abstract | 2 | Background: Oral health is primarily dependent on individual behavior<br>Objective: Analyze the impact of gamification and its mechanisms on oral healthcare.<br>Data sources: Randomized controlled trials and Cross Sectional studies on the effectiveness of gamification in oral health in PubMed and …<br>Study Eligibility criteria: Studies were selected if they were peer reviewed, access to full papers, explains type of research and methods, elected gamification as a research subject, uses gamification elements, reported the effects and outcomes<br>Participants: from all ages<br>Intervention: any form of gamification or serious games<br>Study appraisal and synthesis methods: PICO<br>Results: Oral self-care applications supported by evidence-based oral health was mentioned in 73%  (11/15) of the selected studies. The most clearly defined information's in the applications were "brushing time" 100% (11/11) and "daily amount brushing" 91% (10/11). Most studies, 73% (11/15), mentioned oral healthcare behavior change techniques. Game design elements based on the gamification features for oral hygiene applications were identified in 80% (12/15) of the studies.<br>Limitations: The limited availability of articles related to the main topics and the heterogeneous studies with varying focus and dispersing attention.<br>Conclusions: Gamification in oral healthcare has shown promise as a novel approach for changing people's health behavior. The majority of the studies reported evidence-based oral health and mentioned oral healthcare behavior change techniques. |
| Introduction | | |
| Rationale | 3 | The role of motivation and gamification in human behavior change is coming more evident. What are their applicability and relevance on oral health [illegible] |
| Objectives | 4 | Analising the impact of gamification on oral health and what mechanisms are used. |
| Methods | | |
| Protocol and registration | 5 | None |
| Elegibility criteria | 6 | peer-reviewed; full papers; empirical research (qualitative and quantitative); explained research methods; gamification as subject of study; report of impact; oral healthcare outcomes |
| Information sources | 7 | www.ncbi.nlm.nih.gov; jamir.org; Ovid; www.sciencedirect.com; www.cochranelibrary.com |
| Search Strategy | 8 | See searchstring table |
| Selection process | 9 | 3 reviewers worked independently; 1 worked on PubMed data research; 2 worked on other sources |
| Data colection process | 10 | 3 reviewers collected data from reports, working independently and aggregate information after on google drive files, with exel data spreadsheets, along with the selected articles |

| Section | # | Checklist item |
|---|---|---|
| Data items | 11 | Names of the authors, date of publication, type of study, type of games, gamification mechanisms, number of participants, age of participants, settings, findings |
| Risk of bias assessment | 12 | Each study was evaluated independently by each member of the team, checking randomization, bias on randomized controlled trials |
| Summary measures | 13 | None |
| Sunthesis of results | 14 | |
| Reporting bias assessment | 15 | Quality of evidence in one selected outcome present in studies |
| Results | | |
| Study selection | 16 | Figure 1 (PRISMA flowchart) |
| Study characteristics | 17 | Table 3 |
| Risk of bias in studies | 18 | Table was created for the leveling the bias of the individual studies |
| Results of individ. studies | 19 | Tables with how many people were included, in intervention and in control group, and the estimate |
| Synthesis of results | 20 | |
| Discussion | | |
| Summary of evidence | 21 | The oral health outcomes related to gamification interventions highlighted the role of gamification in promoting oral health care and literacy. It's also evident the limitations of currently available oral healthcare applications. |
| Limitations | 22 | Risk of publication biases, small number of studies on this field of kmoledge, we covered small studies, integrate studies with multiple apps, this review is open to to publication bias and must be corrected in the future |
| Conslusions | 23 | Gamification in oral healthcare does has an impact and the utilization of oral healthcare gamification enhance oral health promotion and literacy. Represents a potential new approach for oral healthcare providers to change people's oral health behavior. The process of mobile health in oral healthcare is in the initial stage. |
| Funding | 24 | None |
| Competing interests | 25 | None |
